# Effectiveness of Pandemic H1N1-2009 Vaccination in Reducing Laboratory Confirmed Influenza Infections among Military Recruits in Tropical Singapore

**DOI:** 10.1371/journal.pone.0026572

**Published:** 2011-10-28

**Authors:** Vernon J. Lee, Chi Hsien Tan, Jonathan Yap, Alex R. Cook, Pei-Jun Ting, Jin-Phang Loh, Qiuhan Gao, Mark I. Chen, Wee Lee Kang, Boon Huan Tan, Paul A. Tambyah

**Affiliations:** 1 Biodefence Centre, Ministry of Defence, Singapore, Singapore; 2 Centre for Health Services Research, National University of Singapore, Singapore, Singapore; 3 Department of Epidemiology and Public Health, National University of Singapore, Singapore, Singapore; 4 National Centre for Epidemiology and Population Health, Australian National University, Canberra, Australia; 5 Department of Statistics and Applied Probability, National University of Singapore, Singapore, Singapore; 6 Defence Medical and Environmental Research Institute, DSO National Laboratories, Singapore, Singapore; 7 Department of Clinical Epidemiology, Tan Tock Seng Hospital, Singapore, Singapore; 8 Headquarters Medical Corps, Singapore Armed Forces, Singapore, Singapore; 9 Department of Infectious Diseases, National University Health System, Singapore, Singapore; Duke-NUS Graduate Medical School, Singapore

## Abstract

**Background:**

Limited information is available about pandemic H1N1-2009 influenza vaccine effectiveness in tropical communities. We studied the effectiveness of a pandemic H1N1 vaccination program in reducing influenza cases in Singapore.

**Methods:**

A surveillance study was conducted among military personnel presenting with febrile respiratory illness from mid-2009 to mid-2010. Consenting individuals underwent nasal washes, which were tested with RT-PCR and subtyped. A vaccination program (inactivated monovalent Panvax H1N1-2009 vaccine) was carried out among recruits. A Bayesian hierarchical model was used to quantify relative risks in the pre- and post-vaccination periods. An autoregressive generalised linear model (GLM) was developed to minimise confounding.

**Results:**

Of 2858 participants, 437(15.3%), 60(2.1%), and 273(9.6%) had pandemic H1N1, H3N2, and influenza B. The ratio of relative risks for pandemic H1N1 infection before and after vaccination for the recruit camp relative to other camps was 0.14(0.016,0.49); for H3N2, 0.44(0.035,1.8); and for influenza B, 18(0.77,89). Using the GLM for the recruit camp, post-vaccination weekly cases decreased by 54%(37%,67%, p<0.001) from that expected without vaccination; influenza B increased by 66 times(9–479 times, p<0.001); with no statistical difference for H3N2 (p = 0.54).

**Conclusions:**

Pandemic vaccination reduced H1N1-2009 disease burden among military recruits. Routine seasonal influenza vaccination should be considered.

## Introduction

Tropical regions experience influenza all year round, usually with two annual epidemic peaks [1] and an impact comparable to temperate regions [2]. In tropical Singapore, seasonal influenza mortality rates have been shown to be similar to temperate and sub-tropical countries [3], and excess deaths in Singapore from previous pandemics were comparable with global estimates [4]. The 2009 influenza pandemic also affected Singapore, infecting 13% of community-living adults during the first epidemic wave [5]. In spite of the clear burden of influenza, seasonal influenza vaccination rates are low for a variety of reasons [3], [5], [6].

Within the national population, closed and semi-closed communities such as schools and militaries have a higher incidence of respiratory infections which can spread to the general population, and are at risk of outbreaks of respiratory infections such as influenza [7]–[10]. However, there have been few influenza surveillance studies showing the effectiveness of influenza vaccines in tropical regions, especially with the 2009 pandemic vaccine in closed and semi-closed communities.

We therefore performed a prospective surveillance study in the Singapore military to determine the incidence of different influenza strains circulating in the military using molecular methods, and evaluate the effectiveness of a targeted pandemic H1N1 vaccination campaign on the incidence of laboratory-confirmed influenza of different subtypes.

## Methods

The Singapore military started a respiratory disease surveillance program in four sentinel camps on 11 May 2009, before the community spread of H1N1 pandemic influenza in late June 2009 [11]. These included one major recruit training camp (camp A), as recruits are especially affected by influenza outbreaks [9], [12]–[14]. All medically fit males in Singapore are conscripted for two years after high school; the servicemen in the recruit camp and other camps were mostly young adults out of high school. These servicemen across all camps live in the camps during weekdays and return home on weekends, maintaining substantial interaction with the general population. All personnel who visited the camps' primary healthcare clinics during the usual consultation hours with febrile respiratory illness (FRI), which we defined as fever≥37.5°C with cough and/or sore throat, were invited to participate. As we intended to capture additional febrile cases that potentially resulted in absenteeism, while excluding milder non-febrile cases, this is slightly different from the usual use of “influenza-like illness” (ILI, fever>38.0°C with cough and/or sore throat). We excluded repeat visits for the same illness episode.

As nasal washes are equally or more sensitive than nasal or throat swabs, and nasopharyngeal aspirates, in detecting influenza [15]–[17], trained healthcare staff obtained nasal washes (from both sides of the nose) from consenting participants. The washes were placed in viral transport media (Copan Diagnostics Inc., Corona, CA, USA) and sent for laboratory testing within 24 hours.

At the study onset, seasonal influenza vaccine was given routinely only to healthcare workers and selected essential personnel in the military. When pandemic H1N1-2009 influenza spread in Singapore, outbreaks were also noted among the military recruits. When the monovalent pandemic vaccine (Panvax H1N1, CSL, Australia) became available in late-2009, all new recruits were vaccinated with this vaccine along with healthcare workers and some other personnel which formed a minority proportion of the non-recruit camp populations.

Written informed consent was obtained from participants, and the study was approved by the military's Joint Medical Committee for Research, and by the institutional review boards of the National University of Singapore and the Australian National University.

### Laboratory Methods

#### Multiplex PCR assays

For all samples, initial testing involved extracting total nucleic acids from each specimen using a RNA extraction kit (Qiagen, Inc, Valencia, CA, USA). Five µl of extract were tested with Resplex II (version 2.0, Qiagen, Inc., Valencia, CA, USA) for respiratory micro-organisms on the LiquiChip 200 Workstation. Resplex II is a multiplex PCR assay coupled with bead array detection technology and can simultaneously detect influenza A and B viruses together with other microbes [18]–[20].

#### Subtyping and sequencing of influenza viruses

Specimens positive on Resplex II for influenza A virus were further subtyped with real-time PCR for H1 or H3 [21], or for pandemic H1N1-2009 [22]. Five µl of total genetic extracts were tested with the one-step SuperscriptIII/Platinum Taq kit (Invitrogen, Carlsbad, CA, USA) on either the LightCycler machine from Roche or the Applied Biosystems real-time PCR machine (7500). Selected specimens were subjected to cDNA synthesis using the uni12primer (AGCAAAAGCAGG) [23] with the Transcriptor First Strand cDNA synthesis Kit (Roche Diagnostics, Mannheim, Germany). PCR amplification of the partial H1 or H3 gene was carried out with primers described by Ghedin et al [24]. Amplicons were sequenced using the same primers and analysed with DNAstar, Lasergene version 7 (Madison, USA). For specimens which were influenza B virus positive, PCR amplification of the partial HA gene was obtained with in-house designed primers (HA471F = 5′ACCTCAGGATCTTGCCCTAACG-3′ and HA1169R = 5′TGTGTATCCGTGCCAACCTGCAAT-3′) and sequenced with the same primers. All sequences were analysed with DNAstar, Lasergene version 7 (Madison, USA).

### Statistical Analysis

Influenza data from the military surveillance were compared to similar published data from the Singapore National Public Health Laboratory which performs surveillance from sentinel primary healthcare sites using ILI criteria. The data are presented on a weekly basis to display temporal variations, while reducing the stochasticity from daily fluctuations and day of week effects [25].

The main intervention was the pandemic H1N1-2009 vaccination program for new recruits from December 2009 onwards using the monovalent Panvax vaccine. We compared rates of laboratory-confirmed influenza in Camp A with the recruit vaccination program to the other camps (B, C and D). Using an intention-to-treat analysis, we did not sub-analyze the small proportion of recruits who were not vaccinated (<1%), individuals in Camps B, C and D who had obtained pandemic H1N1 vaccination outside this specific vaccination program in camp A (19% of the population of Camps B, C and D), or anyone who obtained seasonal influenza vaccine. We used two different statistical methods to minimize possible confounding especially due to potential differences between the camp populations.

In preliminary analyses, we assessed the relative risk of infection by strain in the two groups of camps, Camp A versus the others, by the two time periods, pre- and post- pandemic H1N1 vaccine. We excluded the week during and the week immediately after new recruits entered camp from December 2009 onwards, as vaccination was ongoing. We used a hierarchical model where the probability of infection per week is assumed to follow a beta distribution, with differing mean and variance for each cohort, period and strain. This model was fitted using the Bayesian framework, taking independent, pseudo-objective exponential priors with mean 10,000 for the hyperparameters. Taking a beta distribution for the weekly probabilities results in a distribution that was more diffuse than a binomial with the same mean, and accounts for the non-independence of infectious disease case data in closed populations [26]. We quantified the relative risk via the posterior distribution for the ratio of the means of the beta distribution.

To account for potential confounders in determining the effect of pandemic vaccination on various influenza strains, we subsequently built an autoregressive statistical model to determine the possible association between pandemic vaccination and influenza cases developing post-vaccination. We used a generalized linear model (Poisson with the log-link function) to determine if changes in number of cases for each strain per week were influenced by several key parameters including: number of cases detected by the national surveillance program in the previous week (representing burden of disease outside the camps), number of cases in the camps for the previous week, entry of new recruits on the week of arrival and the subsequent week to account for time taken for vaccination, and a binary indicator variable encoding the recruit camp vaccination program. We assessed for non-linear associations by using polynomial functions of covariates, and terms that were not statistically distinguishable from 0 at the 0.05 level were dropped to obtain the model presented.

Analyses were performed using the R Statistical Software [27] and JAGS version 2.2.0 [28].

## Results

A total of 2,858 subjects with FRI consented to participate from 11 May 2009 to 25 Jun 2010. Their average age was 20.7 years old (SD 3.2), and all but 5 (99.8%) were male. Of these cases, 15% (437), 2.1% (60), and 9.6% (273) were pandemic H1N1-2009, H3N2, and influenza B respectively. There were 1,885 FRI cases from the recruit camp, Camp A, of which 15% (285) were H1N1-2009, 2.1% (39) H3N2, 0.1% (2) seasonal H1N1 and 13% (249) influenza B. There were 973 FRI cases from non-recruit camps, Camps B, C and D, outside the recruit pandemic vaccination program, of which 16% (152) were H1N1-2009, 2.2% (21) H3N2, 1% (8) seasonal H1N1, and 2.5% (24) influenza B. The few seasonal H1N1 cases were excluded from subsequent analyses. There were 43 cases of H1N1-2009 vaccination failure, of whom 27 (63%) had clinical onset less than a fortnight after vaccination.

### Influenza Incidence and Trends

**Figure 1:**
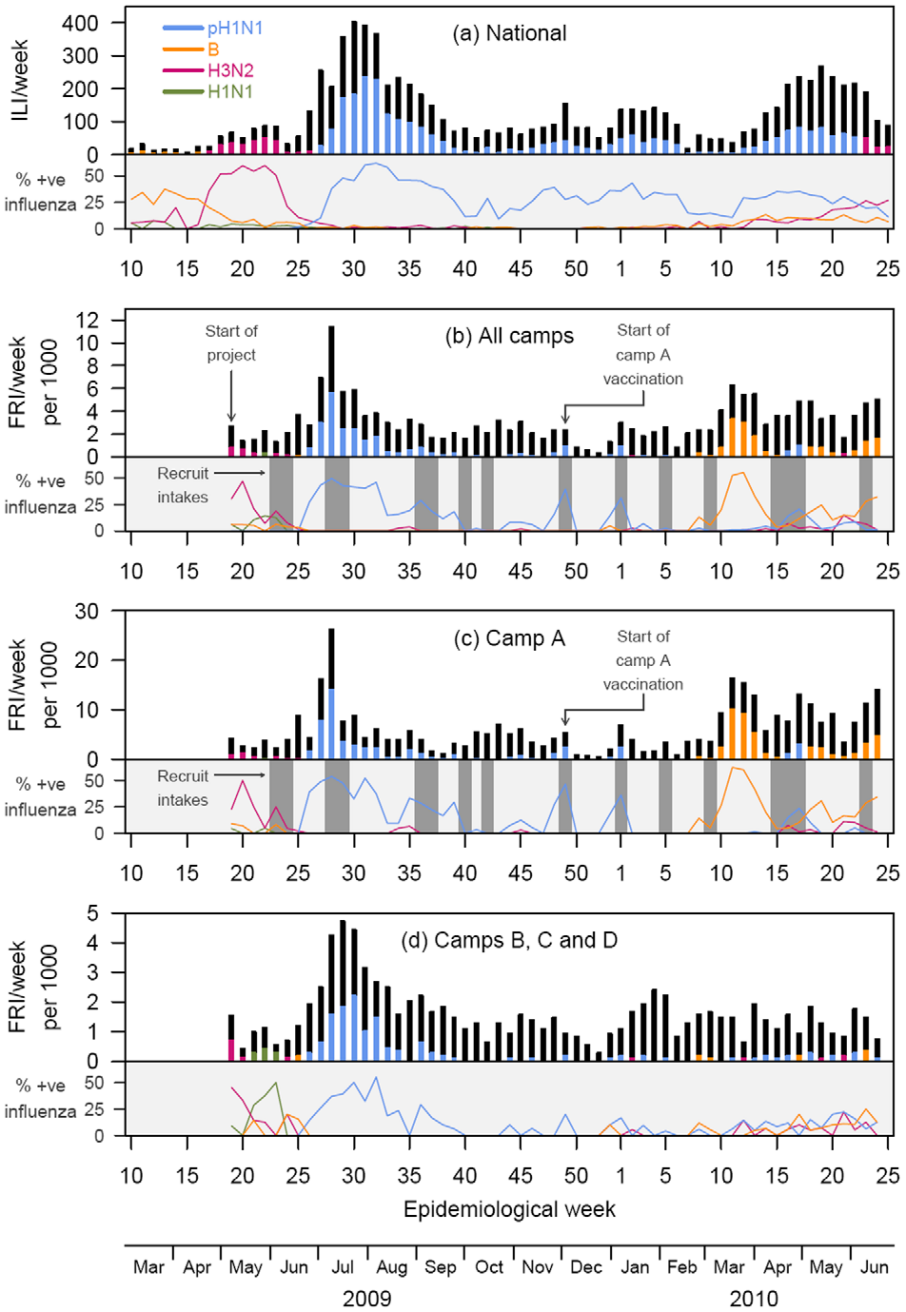
National ILI (a) and Military FRI (b,c,d) Cases by Week, with Influenza Positivity (RT-PCR) by Strain*. *On the white panels, the ILI or FRI cases associated with the dominant strain for the week is colored, while the rest are shaded black. On appropriate grey panels, weeks with recruit intakes are shaded dark grey. Weekly cases of influenza-like illness (ILI, (a), white panel, from national surveillance) and febrile respiratory illness (FRI, (b,c,d), white panels, in military camps) and % positive for influenza by RT-PCR by influenza strain (grey panels, as proportion of all ILI (a) or FRI (b,c,d)). On the white panels, the total number of ILI or FRI cases associated with the dominant strain for each week is colored; for visual acuity, non-dominant strains and influenza negative cases are shaded black. On appropriate grey panels, weeks with recruit intakes are shaded dark grey. Time is measured in epidemiological weeks on each panel; an additional time axis in calendar months is presented at the foot.

The military surveillance data are presented in Figure 1 with national surveillance data as a comparator. A substantial proportion of military cases in May 2009 tested positive for influenza A (H3N2), corresponding to the usual epidemic month [2]. The strain circulating in the military was the A/Perth/16/2009-(H3N2)-like virus which was also circulating nationally.

Soon after local community transmission of pandemic H1N1-2009 on 18 June 2009 [11], the Singapore military reported its first outbreak on 22 June 2009 [29]. Military FRI incidence increased substantially, peaking during the week ending 19 July 09 (about 2 weeks earlier than the general population), with a corresponding growth in PCR-confirmed pandemic H1N1.

The circulation of pandemic H1N1 in the military declined substantially by early October (week 40), corresponding to the end of Singapore's first epidemic wave. However, there were subsequent sharp increases in December 2009 (weeks 48–49), January 2010 (weeks 1–2) and May 2010 (weeks 17–19). The first coincided with the entry of new recruits into Camp A (Figure 1c), with some transmission in the other camps (Figure 1d). This prompted pandemic vaccination for this and subsequent batches of recruits, since the vaccine became available in Singapore by November 2009. Apart from these three outbreaks, pandemic H1N1 incidence among recruits was subsequently very low. In contrast, pandemic H1N1 transmission in non-recruit camps occurred throughout and peaked in May and June 2010 (Figure 1d). For the entire study period, the circulating strain was identified as a A/California/7/2009(H1N1)-like virus, similar to the strain in the 2010 Southern influenza vaccine.

An influenza B outbreak occurred in mid-March 2010 (weeks 11–15), especially among the recruit population; this corresponded to a slight increase in influenza B infection in the general population (Figure 1). The number of cases abated as recruits completed their training, but rose in May 2010 (weeks 18–20) and June 2010 (weeks 23–25) with the entry of new recruits. The circulating strain was identified as being of the Victoria-lineage and closely related to the B/Brisbane/60/2008-like virus, which was the vaccine virus for the 2009/10 Northern and 2010 Southern influenza vaccines.

In May and June 2010, there was also an increase in proportion of H3N2 detected in the general population and the military. The circulating strain was the A/Perth/16/2009(H3N2)-like virus, similar to the vaccine virus in the 2010 Southern influenza vaccine. Seasonal influenza vaccination rates remained low in the military (excluding healthcare workers and essential personnel).

### Vaccine Effectiveness

The estimated relative risk (RR) of pandemic H1N1 infection for Camp A personnel after the vaccination program's start relative to before the vaccination period was 0.056 (95% Bayesian credible interval (BCI): 0.009, 0.16) (Table 1). The RRs in the same period for H3N2 was 0.33 (95% BCI 0.056, 0.95), and for influenza B were 67 (95% BCI 11, 250) respectively. For the other camps, the RRs were 0.50 (95% BCI 0.18, 1.09), 1.2 (95% BCI 0.24, 3.8) and 8.8 (95% BCI 1.0, 39), respectively.

**Table 1:** Relative risks for influenza infection after 2009-H1N1 vaccination program in Camp A, compared to the preceding period, by influenza subtype.

Strain	Camp A (95% BCI)	Camps B,C, and D (95% BCI)	Camp A relative to Camps B,C,D (95% BCI)
Pandemic H1N1	0.056 (0.009, 0.16)	0.50 (0.18, 1.1)	0.14 (0.016, 0.49)
H3N2	0.33 (0.056, 0.95)	1.2 (0.24, 3.8)	0.44 (0.035, 1.8)
Influenza B	67 (11, 250)	8.8 (1.0, 3.9)	18 (0.77, 89)

BCI = Bayesian credible interval.

To compare between Camp A and the other camps, adjusting for different rates of influenza transmission across time, the ratio of the RR of pandemic H1N1 infection for Camp A in the post- to the pre-vaccine periods to the RR in other camps for the same periods (i.e. [risk after H1N1 vaccination in Camp A/risk before vaccination in Camp A]/[risk after H1N1 vaccination in others/risk before vaccination in others]) was 0.14 (95% BCI: 0.016,0.49) suggesting that the vaccination program in Camp A substantially mitigated the risk of pandemic H1N1 over and beyond the reduction observed in other camps without vaccination programs. The corresponding ratio of RR for H3N2 in Camp A relative to other camps was 0.44 (95% BCI 0.035,1.8), while there was a marked growth in the ratio for influenza B of 18 times (95% BCI 0.77,90), although for neither H3N2 nor influenza B was the ratio of RRs statistically discernible from one.

Using the generalized linear model (GLM) for Camp A to account for confounders, we determined that post-vaccination with the 2009 H1N1 pandemic vaccine, the adjusted weekly number of pandemic H1N1 cases significantly decreased by 54% (95% confidence interval (CI): 37%, 67%, p<0.001) from what would be expected without vaccination. Conversely, actual influenza B cases increased by 66 times (95% CI 9 times, 479 times, p<0.001) compared to what would be expected, while no change in H3N2 rates were statistically discernable (p = 0.54).

## Discussion

There is a dearth of information about influenza spread in tropical environments, and how transmission in semi-closed environments such as schools or military camps differs from the general population as high attack rates have been reported [5], [7]. In addition, there is little information about the effectiveness of the pandemic vaccine in tropical regions where a large proportion of the world's population resides, and where early availability and access to such vaccines may pose a challenge.

The pandemic H1N1 outbreaks in the military Camp A in December 2009, January and May 2010, and the influenza B outbreak in March 2010 demonstrate the unique vulnerability of recruit populations. The physical and psychological challenges associated with starting military life, including living in close proximity, can result in increased influenza transmission [30]. In addition, as new recruits enter from the general population, they may introduce infections prevalent in the community, which are then amplified in the closed environment as recruits are initially confined to camp. This probably also applies to other closed and semi-closed communities such as schools and boarding facilities.

Once the vulnerability of the new recruits was recognised and inactivated monovalent pandemic H1N1-2009 vaccine became available, a mass vaccination campaign with the vaccine was introduced from December 2009 for new recruits in Camp A. Our simple Bayesian model showed that the risk for pandemic H1N1 was 86% lower in Camp A than in other camps after vaccine introduction, and similarly the GLM accounting for possible confounders showed a significant decrease of 54% over what was expected in the absence of vaccination. Rates of pandemic H1N1 remained low despite national outbreaks.

Our study provides additional evidence of the effectiveness of monovalent pandemic influenza vaccination. Puig et al showed a 90% vaccine effectiveness in preventing H1N1-associated hospitalisations [31]; Simpson et al, using vaccination status among ILI cases, showed a vaccine effectiveness of 95% [32]. Another multi-centre case-control study in Europe found a lower 66.0% to 71.9% vaccine effectiveness using different statistical computations [33]. Ours is the first study, to our knowledge, conducted in the tropics among a semi-closed population that takes into account the burden of disease both internally and externally to determine the actual effectiveness of the vaccine based on laboratory confirmed infections.

After the introduction of pandemic H1N1 vaccine, we observed a large rise in the risk of influenza B in Camp A compared to the other camps, with a high ratio of RRs of 18 (though with a broad 95% BCI that spanned 1); when accounting for potential confounders and autocorrelation via the GLM, the risk rose to 66 times that in the unvaccinated group, with enough precision to exclude the possibility of no change. Potential reasons could be the dominance of influenza B in the ecological niche of the vulnerable recruits given the effective suppression of pandemic H1N1 by vaccination. Considering that the strain of influenza B responsible for the outbreaks in 2010 was matched to the prevailing trivalent seasonal influenza vaccine, a universal seasonal influenza vaccination program would probably have prevented this.

In addition to providing vaccines to vulnerable populations, the timing of vaccination is important. The two pandemic H1N1 outbreaks in Camp A in January and May 2010 showed that early outbreaks could still occur due to the lag time between vaccination and establishment of immunity. This is substantiated by pandemic H1N1 cases who had been vaccinated within two weeks prior to disease onset which constituted the majority of the “vaccine failures”. One possible solution would be to vaccinate at-risk groups before they congregate: for example, military recruits before they enlist or school children before starting each school year. This may also reduce community outbreaks, since transmission in schools may catalyse community transmission [34].

There are some limitations to this study. As influenza transmission patterns change over time, it is inherently difficult to compare between periods before and after vaccination from time series data, and we relied on statistical tools to provide an inductive estimate of the effect of vaccination. Given the non-independent nature of infectious diseases spread, it is difficult to assess vaccine effectiveness directly with a limited number of cohorts, substantial levels of contact within cohorts, and the distorting signal of the consequent herd immunity. To quantify the uncertainty in our estimates, we used indirect methods that rely on intrinsic replication within the time series data prospectively collected. In addition, the populations of the camps may differ in demographics and on prior exposure to the 2009 H1N1 virus. We used two statistical methods to compare between the camps, and between periods in Camp A to minimize confounding. Studies whose purposes do not include surveillance or assessing group-level vaccine effects might use different designs; with extrinsic replication, either in multiple cohorts or using non-interacting participants, allowing more direct measurements of vaccine effectiveness at the individual level. Finally, we did not survey milder cases without fever and asymptomatic cases, as we had to balance completeness with a focused surveillance program. We have however shown that vaccination reduces febrile cases and therefore the potential impact of the program.

Pandemic 2009-H1N1 vaccine has been effective in reducing the burden of 2009-H1N1 among recruits in the Singapore military, and should be made available early for future pandemics. Seasonal influenza vaccination programs for new entrants to such communities may significantly reduce the impact of influenza further, and should be considered routinely.
